# Supplementary material for: Network control by a constrained external agent as a continuous optimization problem
Source: Sci Rep. 2022 Feb 10;12:2304. doi: 10.1038/s41598-022-06144-4 (PMC8831612; doi:10.1038/s41598-022-06144-4)
Supplement: Supplementary file 1 — Supplementary Information. [file 41598_2022_6144_MOESM1_ESM.pdf]

Network control by a constrained external agent as a continuous optimization problem

Supplementary Information

Jannes Nys[1,2], Milan van den Heuvel[3,*], Koen Schoors[3], and Bruno Merlevede[3]

[1]University of Antwerp - imec, IDLab, Department of Computer Science, Antwerp, 2000, Belgium

[2]Ghent University, Department of Physics and Astronomy, Ghent, 9000, Belgium

[3]Ghent University, Department of Economics, Ghent, 9000, Belgium

[*]Corresponding author: Milan.vandenHeuvel@UGent.be

A Supplementary Figures

In this section, we show figures that fall outside the scope of the main text but are valuable for the reader to gain insight into the flexibility and possibilities the proposed framework provides to study optimal control and the resulting distribution of control and cost in the network.

A.1 Synthetic extended star network

Fig S1-S3 shows more a more detailed view on the distribution of cost and control for the extended star-graph example in the main text. On a case-to-case basis, this can lead to more insights into how both direct and indirect control, and cost is distributed along the network and how it might be connected to other node-specific features (e.g. type of company, centrality).

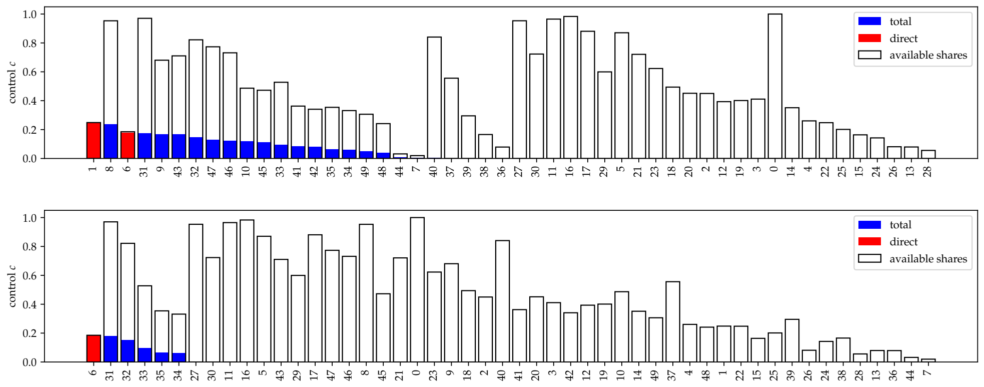

Figure S1: Distribution of control gained at $\lambda = 0.75$ (top) and $\lambda = 1$ (bottom). We show the direct (red) and indirect(blue) control of the external agent for the star network. The companies on the x-axis are sorted in descending order of the total shares bought by the external agent. Total shares available are shown in black. We remind the reader that we artificially set the $o^{\max} = 1$ for root nodes (here company "0" on the x-axis).

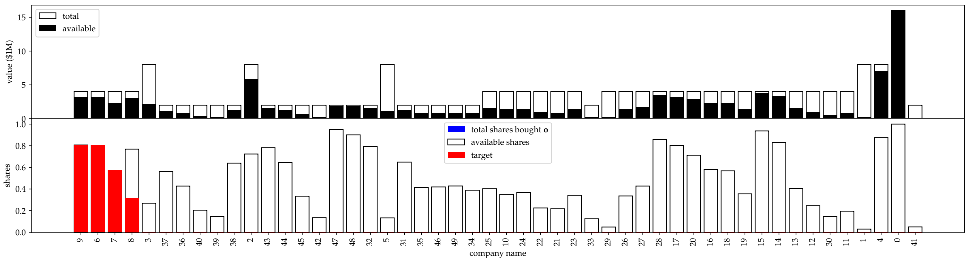

Figure S2: (top) Empty bars represent the total value of the companies in the star network, while the black bars indicate the fraction that is available in the network (determined by the sum of incoming weights). (bottom) Distribution of shares bought in the network by an external agent, where red bars reflect the fact that the considered company is also a target (which is the case for all nodes in the star network). The control distribution is obtained for a budget restriction of €10M.

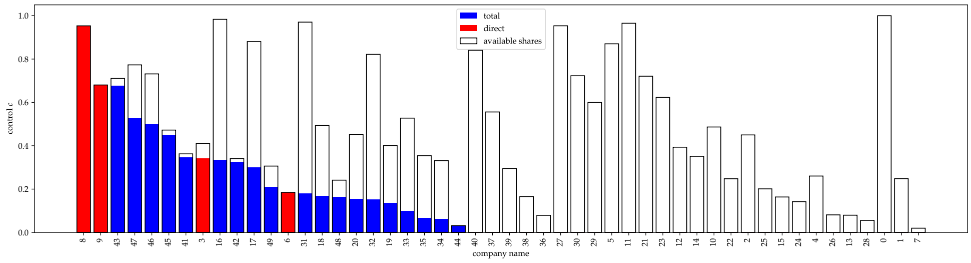

Figure S3: Distribution of control over the companies in the star network. The black bars indicate the total available shares for that company. Blue indicates the total control on a company, of which the fraction of direct control is indicated in red (often the entirety of the bar). The control distribution is obtained for the case in Fig. S2.

A.2 Great Britain biotech research centres

Fig S4 shows the complexity of the relatively small network constructed from the largest connected component of the in-component of the biotech research centres in Great Britain. The obvious complexity of the network shows that automated techniques are needed to gain insights into the structure's effect on control in the network.

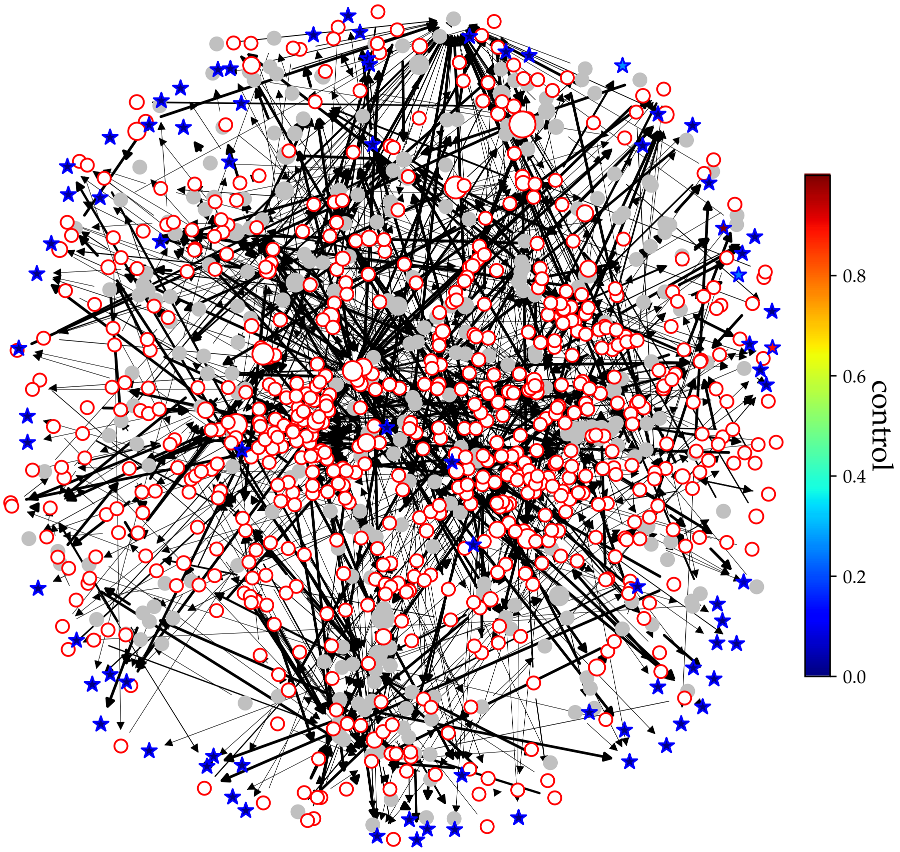

Figure S4: Amount of control (color) over biotech research centres (stars) in Great Britain as targets. The source nodes (red circles) and intermediary companies (grey) are shown for the country constraint policy environment (making all non-GB companies sources, represented by round nodes with red borders). The results are shown for the constrained optimization with a budget that equals total value of all targets. The size of the red circles reflects the cost of acquiring that company in the final result.
